# Time restricted eating as a weight loss intervention in adults with obesity

**DOI:** 10.1371/journal.pone.0246186

**Published:** 2021-01-28

**Authors:** Dunja Przulj, Daniella Ladmore, Katie Myers Smith, Anna Phillips-Waller, Peter Hajek

**Affiliations:** Health and Lifestyle Research Unit, Queen Mary University of London, London, United Kingdom; Weill Cornell Medical College in Qatar, QATAR

## Abstract

**Objectives:**

Time-restricted eating (TRE) is a weight management approach in which food is consumed only within a specific period each day. The simplicity of this approach is appealing, but its efficacy is not known. The aim of this pilot cohort study was to assess adherence to TRE and its effects on weight and lipid profile.

**Methods:**

Fifty participants with obesity attempted to follow TRE for 12 weeks. Surveys were conducted weekly over the phone to assess treatment adherence and ratings; and at 6 and 12 weeks, participants attended the clinic to be weighed, have their blood pressure taken and provide a blood sample for lipid profile. Treatment results were compared with data from previous comparable cohorts using other weight management methods.

**Results:**

Mean age of the participants was 50 (SD = 12.0), mean weight 97kg (SD = 17.1), mean BMI = 35 (SD = 4.0) and most were female (74%). At weeks 6 and 12, 64% and 58% of participants continued to practice TRE on at least five days/week. Using the ‘last observation carried forward’ imputation, mean (SD) weight loss was 2.0 (1.7) kg and 2.6 (2.6) kg at 6 and 12 weeks. Among participants who provided follow-up data, those who adhered to the intervention for at least five days/week recorded greater weight loss than those with lower adherence (week 6: 2.5 (1.7) vs 1.0 (1.3), p = 0.003; week 12: 3.5 (2.7) vs 1.3 (2.0), p = 0.001). A total of 26% of the sample lost at least 5% of their body weight at 12 weeks. The intervention had no effect on blood pressure or lipid profile.

**Conclusions:**

TRE results were modest, but at least on par with those achieved with more complex interventions, and weight loss did not decline at 12 weeks. A formal trial of the intervention is warranted.

## Introduction

Over a quarter of adults in England are living with obesity, with prevalence being higher in disadvantaged socioeconomic groups [1]. Ill health resulting from obesity is considered to be responsible for about 10% of morbidity and mortality in the UK [2]. Simple and effective weight loss interventions that are easy to disseminate are lacking. Intermittent fasting (IF), involving periods of total or partial energy restriction, alternated with non-restricted energy intake, is one potentially promising approach.

There is preclinical evidence showing that in animals, regular periods of IF accompanied by no other food restrictions, generates weight loss and improvements in cardio-metabolic health [3–6]. In humans, IF was shown to generate weight loss [7–18] as well as improvements in glucose metabolism and lipid profiles [8, 9, 12, 14–16, 18].

In terms of clinical practice, however, the approach has a notable limitation; the existing human studies typically used a demanding form of IF, with fasting every other day [7–18], usually with caloric restrictions during non-fasting days [9, 12, 13, 16]. The studies provided food free of charge for at least some of the time, and participants were carefully selected—in some studies they had to pass a pre-test of energy restriction prior to randomisation [8, 9]. Within tightly controlled trials, a proportion of selected clients were able to persist with this approach for the trial duration, but the approach seems too difficult for most people and it is not used in clinical practice.

IF, however, can be also implemented in less severe formats. ‘Time-Restricted Eating’ (TRE) or ‘16:8’ diet requires dieters to consume food only within a specific ‘window’ each day, typically over eight hours, and refrain from any eating outside of this window.

Animal studies suggest that even in this much simplified format, the diet may still convey benefits [3, 6, 19]. Several small cohort studies have assessed effects of TRE on weight in humans: TRE practiced by 11 participants over 4 days reduced swings in hunger and increased fat and protein oxidization [20]; weight loss of some 3kg was reported in 8 participants who adhered to a 10–12 hour eating window for 16 weeks [21]; of 23 participants instructed to eat only between 10am and 6pm for 12 weeks, 16 remained in the study and reported adherence on 5–6 days per week and lost around 3kg [22]; more recently, 19 participants who trialled a 10-hour eating period for 12 weeks, lost on average 3.3kg [23]. In one small trial, seven participants randomised to TRE for 10 weeks, found adherence challenging and only lost 0.7 kg. There was no difference in weight change compared to controls who continued with normal eating patterns [24].

Two cross-over studies have examined the effects of the timing of TRE. In a laboratory-based experiment where food was provided, 8 pre-diabetic men who attempted ‘early’ TRE (9am - 3pm) for 5 weeks, lost on average 1.4kg vs. 1kg when eating over a 12-hour period [25]. They also reported a reduced desire to eat in the evenings. The second study compared early TRE to late TRE [26]. Fifteen men with obesity were asked to follow two variations of TRE for 7 days each; one with an eating period of 8am-5pm and the other 12pm-9pm. There were no significant differences in weight loss between the two conditions (1.3kg for early vs. 0.8kg for late).

Religious fasting practices also provide an indication of TRE effects, e.g. a cohort adhering to Ramadan fasting lost on average 1kg of weight, but this was regained within a month [27]. A recent review [28] that included both TRE trials and studies of Ramadan fasting, drawing mostly upon Ramadan fasting studies, reported greater weight loss with TRE compared to control groups without time restrictions (weighted mean difference: -1.07 kg, 95%CI: -1.74 to -0.40; p = 0.002).

The studies to date have been small and eating time-periods have varied between studies, from 6 to 12 hours, with some allowing participants to choose their preferred eating period and others not. Due to small samples and varied results, information is limited on the size of weight loss that TRE can be expected to generate. We conducted a larger study to assess ‘real life’ adherence to TRE and its effects on weight and lipid profile, in participants with obesity seeking help to lose weight.

## Methods

### Design

This was a cohort study that followed up participants over 12 weeks.

### Participants

Fifty-two participants seeking to lose weight were recruited during February- June 2018, primarily through our community weight clinic and via adverts, the University staff bulletins and social media. Follow-up was completed in September 2018.

Participants were eligible if they were aged 18 years or over and had a BMI over 30kg/m^2^, or over 28kg/m^2^ with co-morbidities. Participants were not eligible if they had any medical condition precluding fasting, including a history of eating disorders; any serious illness; were currently on psychiatric medication; were pregnant or breastfeeding; had lost more than 5% of body weight in the previous 6 months; or were currently using TRE or another fasting approach for weight loss.

### Procedures

Prospective participants contacted the study team by telephone or e-mail and were pre-screened for eligibility. Eligible participants were invited to a baseline visit at Queen Mary, University of London.

At the baseline visit, written consent was collected, and participants’ eligibility was confirmed. Eligible participants completed study questionnaires (see below), measurements of weight and blood pressure were taken, and a blood sample for lipid profile was collected. Participants then individually received an explanation of the TRE intervention by a member of the research team with experience in weight management. The session took approximately 30 minutes.

Participants attended the study centre one and six weeks later to be weighed and to discuss their progress. They also received phone calls at weeks 2, 3, 4 and 5, that monitored progress and collected data on adherence and other ratings, detailed below.

At the final visit at 12 weeks, measures of weight and blood pressure, and a blood sample for lipid profile were taken, and participants reported on TRE adherence and provided intervention ratings.

Participants received £10 at the 6 and 12-week visits as a compensation for their time and travel. Participants who agreed to provide a blood sample at baseline and 12 weeks received an additional £10 for each sample.

Written informed consent was obtained from participants. The study was approved by Queen Mary Ethics of Research Committee, ref: QMERC2017/71. The trial was registered on ISRCTN, ref: ISRCTN16400313.

### TRE intervention

Participants were asked to eat only during an 8-hour period each day for the next 12 weeks. Over the remaining 16 hours each day, participants could drink water, diet drinks or coffee/tea with no milk or sugar. Participants were free to choose the 8-hour period most convenient for them and were instructed to start TRE the following day.

A leaflet was provided explaining the TRE intervention and providing advice on choosing a suitable time frame and on coping with hunger, information on beverages that could be consumed during fasting, and details of study procedures (see S1 Appendix)

Participants were given a diary card to keep track of their TRE adherence and hunger ratings.

### Measures

Demographic data were collected at baseline. Weight was measured at baseline and at 1, 6 and 12 weeks. Blood pressure and lipid profile were assessed at baseline and at 12 weeks. When a high blood pressure reading was recorded, three readings were taken over the course of 5 to 10 minutes and the mean measure was recorded.

TRE adherence was assessed by asking participants each week, on how many days they had completed TRE and reasons for any non-adherence were noted. Ratings of TRE included helpfulness (5-point scale from 1 = ‘not at all’ to 5 = ‘extremely helpful’); how difficult it was to adhere to TRE since the last contact; how hungry the participants were on days they adhered to TRE since the last contact (both items rated from 1 = ‘not at all’ to 10 = ‘extremely’); whether participants ate more or less food than usual compared to before starting TRE (with the options: more than before, the same, less than before, not sure); whether there was a part of the day they felt more hungry or uncomfortable (morning, afternoon, evening, at night, no clear difference); and how likely they were to complete TRE daily for the next 7 days (1 = not at all likely to 10 = extremely likely). The 8-hour time periods that the participants selected were also recorded. At week 12, participants were asked to rate how likely they were to carry on with TRE once the study was over and how likely they are to recommend the approach to a friend (both items rated from 1 = not at all likely to 10 = extremely likely).

At weeks 1 and 6, open-ended questions asked participants to list any barriers and facilitators to TRE, and at week 12, participants were asked whether they would recommend TRE to friends (1 = definitely not to 10 = definitely yes).

Blood samples were analysed by The Doctors Laboratory Ltd., London, UK for low density lipoprotein (LDL), high density lipoprotein (HDL), total cholesterol and triglycerides. Participants were not required to fast prior to blood tests [29] or blood pressure check.

### Outcomes

The primary outcome was adherence to TRE. Secondary outcomes included: drop-out rates during the follow-up period, weight change; changes in blood pressure and lipid profile from baseline to 12 weeks; ratings of the intervention; huger ratings; preferred eating time-periods that participants selected; and barriers and facilitators to the intervention.

Drop-out rates, adherence levels and weight change were compared with those in our previous trial that recruited 300 comparable participants from the same geographical area [30]. The trial provided data on weight loss achieved at 12 weeks with standard NHS weight-loss advice, the 5:2 diet accompanied by group support (5:2G) and 5:2 diet provided via one-off brief advice (5:2SH). The Standard Advice and 5:2SH conditions were comparable in contact time to the TRE intervention in this trial.

Finally, the study also aimed to estimate the effect size for a future randomised trial, if TRE was deemed feasible and the observed weight loss was encouraging.

### Statistical analysis

Descriptive statistics were used to report TRE adherence (including exact binomial 95% CI’s) and drop-out rates; changes in weight at weeks 6 and 12; preferred eating periods; time of day most hungry, and food intake over time. Barriers and facilitators to completing TRE were categorised and reported descriptively.

One-way ANOVA (or Mann-Whitney U test for non-parametric data) was used to compare weight change in ‘adherers’ and ‘non-adherers’. In calculating weight change, two imputations were used, last observation carried forward (LOCF) and baseline observation carried forward (BOCF). BOCF is a stricter outcome that assumes that participants lost to follow-up lost no weight.

Paired-samples t-tests (or Wilcoxon signed-rank test for non-parametric data) assessed changes over time in lipid profile, blood pressure, ratings of TRE helpfulness, difficulty adhering to TRE, likelihood of continuing with TRE and hunger.

Cohen’s d imputation was used to estimate the effect size of TRE compared to a previous cohort of weight participants following standard brief advice. The sample size required for a future randomised trial was also calculated.

All data were analysed using SPSS v25. The full dataset is available in ‘S1 Data’.

In this early phase exploratory research, we opted for a pragmatic sample size of 50, achievable economically and quickly, but large enough to allow an estimate for the sample size for a future randomised trial, should the intervention generate an effect, and provide reasonable confidence intervals on key estimates based on 95% CI width (e.g. if 50% adherence rate is observed at 12 weeks, the 95% exact CI width would be 28%. Previous studies reported a weight loss with TRE of 3kg (SD = 3). If this was found in this study, the 95% exact CI width would be 1.7 (SD = 3), or 1.1 if SD = 2).

## Results

### Participants

**Fig 1:**
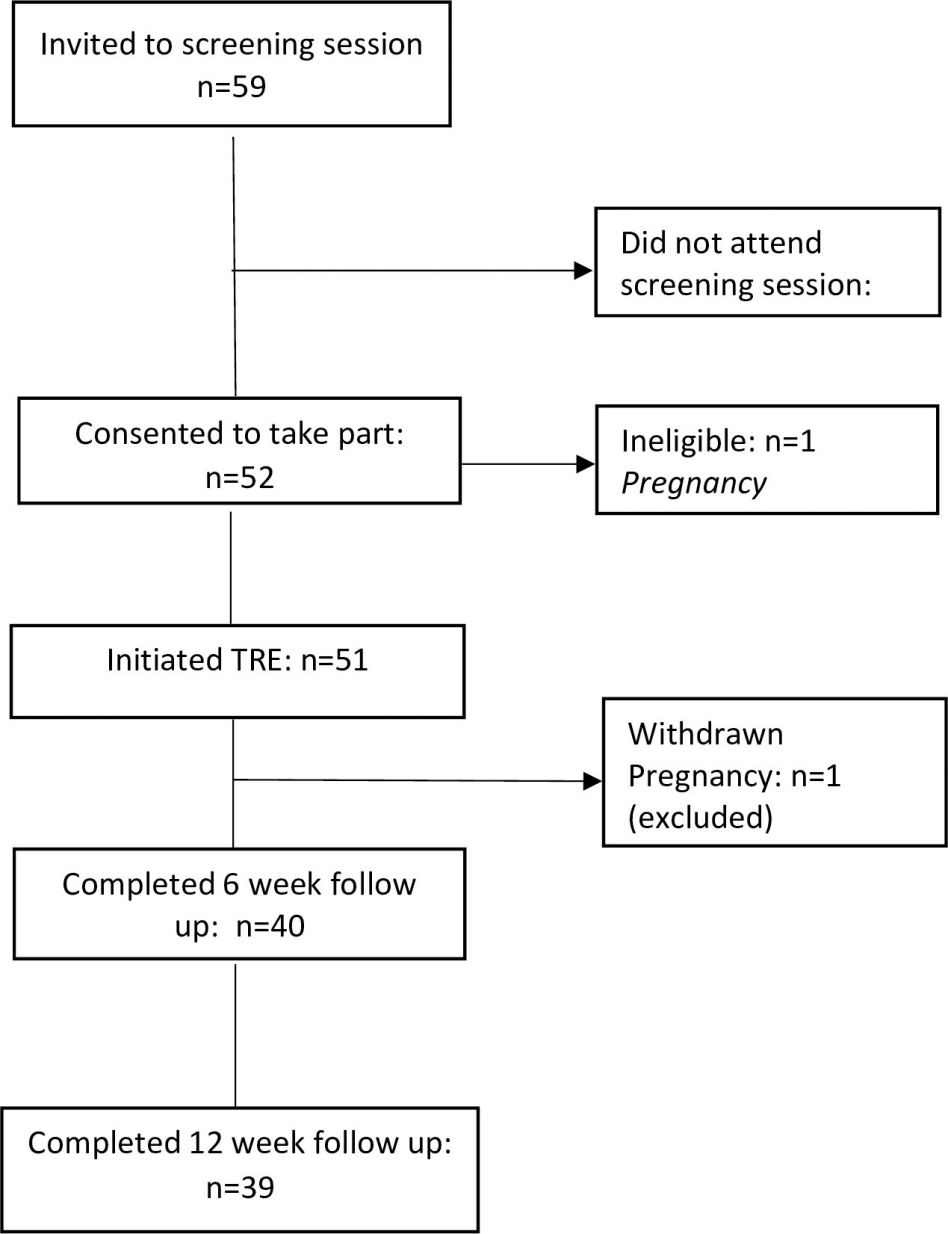
Participant flow.

Of 52 participants consented, 51 initiated the TRE intervention (one participant became ineligible due to pregnancy). During the study, one participant was withdrawn due to pregnancy, leaving a total of 50 participants included in the analysis. Participant flow throughout the study is shown in Fig 1.

Participants were predominantly female, normotensive, with a mean BMI of 35.2 kg/m^2^ (see Table 1).

**Table 1:** Sample characteristics (n = 45–50)*.

Female n (%)	37 (74)
White British n (%)	18 (36)
Education to degree level or equivalent n (%)	30 (60)
In paid employment n (%)	37 (74)
Mean age (SD)	50.1 (12.0)
Mean Weight (Kg) (SD)	97.2 (17.1)
Mean Body Mass Index (SD)	35.1 (4.0)
Mean BP (mmHg)(SD)	119.0 (25.4)/80.6 (24.9)
Number of past serious weight loss attempts (SD)	6 (5.6)

*N varies due to missing data.

### Adherence to TRE intervention

A total of 40 (80%) participants provided data at 6 weeks and 39 (78%) at 12 weeks (one follow-up was completed over the phone at each of these two time points).

Among the participants who provided data, TRE was completed, on average, on 5–6 days per week throughout the 12-week period (see Table 2).

**Table 2:** Mean number of days on which TRE was completed.

	Week 1 N = 47	Week 2 N = 39	Week 3 N = 39	Week 4 N = 37	Week 5 N = 32	Week 6 N = 40	Weeks 7–12 N = 39
**Days (SD)**	5.2 (1.8)	5.3 (1.8)	5.5 (1.6)	5.0 (2.2)	5.6 (1.6)	5.7 (2.0)	5.1 (2.4)

At week 12, 29 (58%, 95%CI 43%-72%) participants continued to adhere to TRE on at least 5 days/week.

### Weight change

Table 3 shows change in weight in the full sample and in TRE adherers and non-adherers. Participants who reported adhering to TRE on at least 5 days/week lost more weight than those who did not. A total of 13 participants (26%) lost at least 5% of their body weight at 12 weeks.

**Table 3:** Change in weight at 6 and 12 weeks.

	All (N = 50) (95%CI)	Adherent*	Non-adherent	Difference** (95%CI)
	Mean Kg (SD)			
BOCF Week 6	-1.9 (1.7) (1.4–2.4)	-2.5 (1.7) N = 32	-0.9 (1.4) N = 18	p = 0.002 (1.4–2.4)
BOCF Week 12	-2.5 (2.7) (1.7–3.3)	-3.5 (2.7) N = 29	-1.1 (2.0) N = 21	p<0.001^ (1.7–3.3)
LOCF Week 6	-2.0 (1.7) (1.5–2.6)	-2.5 (1.7) N = 32	-1.0 (1.3) N = 18	p = 0.003 (1.5–2.5)
LOCF Week 12	-2.6 (2.6) (1.8–3.3)	-3.5 (2.7) N = 29	-1.3 (2.0) N = 21	p = 0.001^ (1.8–3.3)

*Adhered to TRE on at least 5 days every week.

**Adherent vs. Non-adherent.

^ Mann-Whitney U Test.

BOCF = Baseline observation carried forward; LOCF = Last observation carried forward.

### Change in lipid profile and blood pressure

Lipid results from both baseline and 12 weeks were available for up to 31 participants. There were no significant changes in lipid profiles between baseline and 12 weeks (see Table 4). The study also compared changes in the lipid profile in participants who lost at least 5% of their body weight (N = 9) and those who did not (N = 21). LDL reduced from baseline to 12 weeks in those who lost 5% of body weight and increased in those who did not (-0.3 vs. +0.2, p = 0.04).

**Table 4:** Lipid profile at baseline and at 12 weeks (n = 31).

	Baseline	12 weeks	Difference
	Mean (SD)		
Total chol. mmol/L	5.2 (0.9)	5.2 (1.2)	p = 0.539
LDL mmol/L (N = 30)*	2.8 (0.8)	2.9 (1.0)	p = 0.148
HDL mmol/L	1.4 (0.4)	1.4 (0.4)	p = 0.514
Triglycerides mmol/L	2.3 (1.2)	2.0 (1.0)	p = 0.065**

Optimal ranges: Total chol ≤5mmol/L; LDL ≤3mmol/l; HDL≥1mmol/L; triglycerides ≤2.3 mmol/L.

*baseline value could not be obtained for 1 participant.

**Wilcoxon signed-rank test.

There were no changes in blood pressure (systolic BP = 130.2 (SD = 17.8) vs 129.4 (SD = 20.1);p = 0.712 and diastolic BP = 80.7 (SD = 11.2) vs 80.6 (SD = 13.2); p = 0.929 at baseline and 12 weeks, respectively) and no differences in blood pressure changes between participants who lost at least 5% of their body weight and those who did not.

### Ratings of the TRE intervention

Table 5 shows the mean ratings of the intervention by participants who provided data at all three time-points. TRE was rated as moderately helpful throughout this time period. Adhering to TRE became somewhat easier over the first 6 weeks, but readiness to carry on with the programme diminished. A total of 24 (60%) participants rated the likelihood that they will carry on with TRE after the trial ended as high (ratings of 7 to 10), while 27 (69%) rated the likelihood that they recommend the approach to a friend as high (ratings of 7 to 10).

**Table 5:** Ratings of the TRE intervention at different time points.

	Week 1	Week 6	Week 12	Difference*
	Mean (SD)			
Helpfulness (1–5) (N = 32)	3.1 (1.2)	3.4 (1.2)	3.0 (1.2)	Wk1-Wk6: p = 0.134
				Wk1 = Wk12: p = 0.788
Difficult to do (1–5) (N = 33)	3.7 (1.8)	3.0 (2.1)	3.3 (2.4)	Wk1-Wk6: p = 0.032
				Wk1-Wk12: p = 0.248
Likely to carry on (1–10) (N = 35)	9.3 (1.4)	8.7 (2.0)	6.6 (3.4)	Wk1-Wk6: p = 0.113
				Wk1-Wk12: p<0.001
Would recommend to friend (1–10) (N = 39)	-	-	7.6 (3.5)	-

*Wilcoxon signed-rank test.

### Changes in hunger

Ratings of hunger from participants who provided relevant data at all time points (n = 34) indicate a reduction in hunger from week 1 (Mean (SD) = 4.2 (1.7) to week 12 (Mean (SD) = 3.6 (2.1); p = 0.032). Hunger at week six (Mean (SD) = 3.7 (2.2)) was also lower than at week 1, but the difference was not significant (p = 0.071).

### Preferred eating period

Around half of the participants opted for starting their 8-hour eating period between 12pm and 2pm (48%, 52% and 55% at 1, 6 and 12 weeks, respectively). The period between 10am and 12pm was the second most popular (42%, 32% and 32% at 1, 6 and 12 weeks, respectively). Only a few participants opted for earlier or later periods.

As most participants delayed the first meal of the day, a higher proportion of participants reported feeling most hungry in the mornings than at other times (46%, 49% and 40% at weeks 1, 6 and 12, respectively).

Of the 37 participants who provided feedback, only four (11%) reported that compared to their normal eating habits, they increased food intake during the TRE eating periods.

### Barriers and facilitators

Regarding the open questions about factors that helped and hindered TRE adherence, 43 participants provided feedback at different time points. The most common barriers were social occasions such as dining out, having visitors and having drinks after work. These barriers tended to increase over time. In contrast, problems with planning and adhering to TRE over weekends decreased over time.

The most common facilitators were planning in advance and coping with hunger by using distraction or drinking water or black coffee.

### Comparison with previous trial

Table 6 shows weight loss in this and in the previous study [30]. TRE achieved results similar to those seen with a brief 5:2 intervention and with standard multimodal advice.

**Table 6:** Weight loss with TRE and with other interventions.

	Baseline weight (kg)	% available for 12 week FU	LOCF Weight loss at 12 weeks (kg)	% losing ≥5% body weight at 12 weeks	% still adhering to Intervention at 12 weeks
TRE (N = 50)	97.2	78	-2.6	26	58
5:2SH (N = 100)	94.8	77	-2.8	24	58
Standard advice (N = 100)	98.0	71	-1.9	15	49

### Estimating sample size for future trial

Table 6 provides information that can be used to power a future trial. An estimate of effect size comparing TRE and standard advice suggests a small effect (*d =* 0.23), but this could still be worthwhile, especially if the groups further diverge over longer time periods. To have 90% power to detect a difference between weight loss achieved with TRE and that achieved with standard brief advice at12 weeks, (2.6 kg vs 1.9kg (SD = 3.0), alpha level = p<0.05, double sided, using an independent t-test), N = 387 participants per study arm will be required (total N = 774). Future trials, however, will be more informative with a longer follow-up.

## Discussion

Time restricted eating was well tolerated and adherence to the programme over the course of 12 weeks was relatively high. Weight loss was modest, but at least on par with that achieved with other brief interventions that are more complex.

The results concerning participants’ rating of the programme and their adherence to the plan are encouraging. Hunger during the fasting periods was rated as relatively low, and it further subsided with time, suggesting a degree of habituation. Most participants found the programme manageable and would recommend it to friends.

In contrast to results reported in animal studies, TRE had no clear impact on lipid profile, but these measurements were close to or within normal ranges at baseline. There was also no effect on blood pressure, which tallies with a meta-analysis of data from weight management trials that found no association between weight loss and change in blood pressure [31].

Participants who adhered to TRE for the full 12 weeks lost more weight than those who did not, but it is of interest to note that those who did not manage full adherence also lost weight. An earlier pilot reported that participants adhering to TRE recorded a calorie deficit of 300 k/cals per day [22]. Even partial adherence may generate a degree of weight loss over time.

The magnitude of weight loss was similar to that reported in previous smaller cohort studies [21–23]. It was also at least on par with that found in our previous randomised trial that compared standard weight loss advice (comprising information on exercise, self-monitoring, calories and healthy eating) with the 5:2 diet delivered via a one-off session and a written guide. Both of these conditions provided a similar level of support to the support that participants received in the current study. In this context, TRE results look encouraging.

The study has several limitations. There was no control group and the comparison with previous cohorts needs to be interpreted with caution. Randomised trials are needed to establish the potential of TRE in weight management. The sample size was relatively small, and the key estimates of adherence and weight loss have relatively large confidence intervals. Ratings of hunger were measured using a single scale designed for the study, but similar single-scale ratings of hunger were shown to be sensitive e.g. to attentional focus [32] and preferred food [33]. The sample comprised mostly of women, as is typical for weight loss interventions.

The crucial question that the present study could not answer is whether the TRE regime is sustainable over longer periods of time. The majority of participants were able to sustain the programme for three months. The simplicity of the approach and benefits that the participants reported suggest that the regime may be more sustainable than standard approaches that are typically more complex and that generate more discomfort and require more sustained self-control. Weight loss did not decrease over the study period. This contrasts with weight regain that is typically observed with standard weight loss approaches. With the caveat that the follow-up period was only 12 weeks, the observation raises the possibility that benefits may continue to accumulate over longer time periods. On the other hand, participants still reported a degree of hunger during the fasting periods, and adherence declined somewhat between six and 12 weeks. It is thus also possible that the rate at which dieters abandon the programme may be similar to that observed with other approaches, and that long-term effects may be small.

The study generated new information on preferred fasting periods and strategies for coping with hunger that could make the approach more palatable and productive for dieters in the future. Most participants delayed the first meal of the day and experienced a degree of discomfort during the long mornings without food. Participants also reported that the main barriers to TRE were social occasions. If the fasting period was shorter and ‘days off’ were permitted, this could be expected to reduce short-term weight loss; but if it made the intervention sustainable for more people and over a longer period of time, it could increase its benefits overall.

## Conclusions

In summary, the results of this early study are encouraging enough to suggest that a randomised trial with long-term follow-up is warranted.

## Supporting information

S1 Appendix Participant instructions for TRE. (DOCX)

S1 Data Full study data set. (SAV)

S1 Checklist CONSORT 2010 checklist of information to include when reporting a randomised trial*. (DOC)

S1 Protocol (DOC)

S2 Protocol (DOC)
