# Supplementary material for: Time restricted eating as a weight loss intervention in adults with obesity
Source: PLoS One. 2021 Jan 28;16(1):e0246186. doi: 10.1371/journal.pone.0246186 (PMC7842957; doi:10.1371/journal.pone.0246186)
Supplement: S1 Appendix — (DOCX) [file pone.0246186.s001.docx]

**Time restricted eating (TRE)**

**This guide explains time restricted eating (TRE) and how to incorporate it into your daily life. Please take the time to read this information in full.**

**HOW TO DO TRE**

TRE or the 16/8 diet is simple. You only eat for 8 hours a day and do not eat at all during the rest of the day (and night).

The 8-hour period in which you choose to eat is up to you. Some people delay the first meal until mid to late morning so that you can continue to eat and drink as normal into the evening, others feel they need their breakfast and so stop eating early afternoon. For example, if you have breakfast at 7:30 am, the last meal you could eat in the day would be at 3:30pm. If you skip breakfast and have your first bite at lunchtime at 12, you can eat up to 8 pm.

It might take you a few days to work out which time period works best for you, but once you have your schedule, it is a good idea to stick with it. You will then gradually adjust to it and will experience little discomfort during the period when you don’t eat.

You will sometimes need to adjust the schedule to fit in with other plans, and there may be days when you break the rule. This is fine as long as you get back to TRE as soon as you can. We are also asking you to record this on your diary card (this is for the study purposes, rather than for us telling you off!).

If you decide to stop TRE, please do let us know. As with all research, we would like to know when things don’t work or help, as well as when they do.

During the time outside your 8-hour window, you can of course drink, but not beverages that contain calories. Water, black coffee, black tea, herbal/flavoured tea and diet drinks are all fine.

**DEALING WITH HUNGER**

It may take a few days or weeks to get used to TRE. During the early period, you may feel hungry at times when you would previously eat. If you are feeling hungry towards bedtime, having a warm drink such as a flavoured tea and having an early night can help. If you are feeling hungry in the morning, it may help to remind yourself that you are only delaying the meal and that you will be eating soon. You are likely to enjoy the meal more than usual as well. Black coffee can alleviate hunger.

A general advice for coping with hunger is to remind yourself that this means that your body is burning fat; and to keep your mind occupied with something else.

If you are feeling unwell as a result of hunger then do eat, but we ask that you keep a note if this happening and report this back to the study team at the next follow up.

**STUDY PROCEDURE**

Below is a reminder of what you can expect to happen over the next 12 weeks.

| Week 1 | *You will* ***attend the clinic,*** *and we will check how your first week on TRE has been going. You will complete a questionnaire and we will take your weight.* |
| --- | --- |
| Weeks 2-5 | *We will* ***call you each week*** *to see how you have been getting on with TRE. We will also ask you to tell us your current weight.* |
| Week 6 | *You will* ***attend the clinic*** *and we will check on your progress with TRE. You will complete a questionnaire, and we will take your weight.* |
| Week 12 | *You will* ***attend the clinic*** *and we will check on your progress with TRE. You will complete a questionnaire, and we will take your weight, blood pressure, and a blood sample.* |

We will also provide you with a card for each week to record your daily hunger rating and whether you have completed TRE. We will collect the cards at your week 6 visit. At weeks 2 -5 we will ask you about TRE over the phone, so it will be good to have the card at hand when we call.

An example of how to complete the diary is given below.

|  | **Mon** | **Tues** | **Weds** | **Thurs** | **Fri** | **Sat** | **Sun** |
| --- | --- | --- | --- | --- | --- | --- | --- |
| Completed TRE | Yes | Yes | Yes | Yes | Yes | Yes | NO |
| Eating Times | 10 to 6 | 11 to 7 | 10:30 to 6:30 | 12 to 8 | 12 to 8 | 12 to 8 | 8to 7 |
| Hunger Rating | 5 | 5 | 6 | 3 | 8 | 7 | 5 |
| What was your biggest meal? | B | D | D | D | L | S | S |

Hunger Rating: 1 = not at all hungry and 10 = extremely hungry

Biggest Meal: B= Breakfast L= Lunch D= Dinner S= All the same

If you do not complete TRE on any given day then do not worry. Record ‘No’ on your card, note the ‘eating times’ as usual even if they span more than 8 hours, and try to continue with TRE the next day.

If you have any concerns or questions throughout the study then please do let us know on 0207 882 8230 or email [health-research@qmul.ac.uk](mailto:health-research@qmul.ac.uk)

**Thank you for your help with this important study!**
