# Supplementary material for: Time restricted eating as a weight loss intervention in adults with obesity
Source: PLoS One. 2021 Jan 28;16(1):e0246186. doi: 10.1371/journal.pone.0246186 (PMC7842957; doi:10.1371/journal.pone.0246186)
Supplement: S1 Protocol — (DOC) [file pone.0246186.s004.doc]

**Study Protocol**

# TITLE PAGE

**Full Title** Time restricted eating as a weight loss intervention in obese adults: A pilot study.

**Short Title/Acronym** TRE

**Sponsor** Queen Mary University of London (QMUL)

Contact person of the above sponsor organisations is:

Sally Burtles

Head of Research Resources

Joint Research Management Office

5 Walden Street

London

E1 2EF

Phone: 020 7882 7260

Email: [sponsorsrep@bartshealth.nhs.uk](mailto:sponsorsrep@bartshealth.nhs.uk)

**REC Reference/IRAS number** QMERC2017/71

**Chief Investigator** Dr Dunja Przulj, Health and Lifestyle Research Unit, Wolfson Institute of Preventive Medicine, Queen Mary University of London

**Sites** 1. Queen Mary University of London

Health and Lifestyle Research Unit

2 Stayner’s Road

London

E1 4AH

# GLOSSARY of Terms and Abbreviations

ASR Annual Safety Report

BMI Body Mass Index

CA Competent Authority

CI Chief Investigator

CRF Case Report Form

CRO Contract Research Organisation

GAfREC Governance Arrangements for NHS Research Ethics Committees

GCP Good Clinical Practice

HRA Health Research Authority

ICF Informed Consent Form

IPAQ International Physical Activity Questionnaire
JRMO Joint Research Management Office

NHS REC National Health Service Research Ethics Committee

NHS R&D National Health Service Research & Development

Participant An individual who takes part in a clinical trial

PI Principal Investigator

PIS Participant Information Sheet

QA Quality Assurance

QC Quality Control

REC Research Ethics Committee

SOP Standard Operating Procedure

TMG Trial Management Group

TRE Time Restricted Eating

# SIGNATURE PAGE

**Chief Investigator Agreement**

**The clinical study as detailed within this research protocol (Version 1, 6^th^ October 2017,), or any subsequent amendments will be conducted in accordance with the Research Governance Framework for Health & Social Care (2005), the World Medical Association Declaration of Helsinki (1996) and the current applicable regulatory requirements and any subsequent amendments of the appropriate regulations.**

**Chief Investigator Name:** Dr Dunja Przulj

**Chief Investigator Site:** QMUL

**Signature and Date:**

**
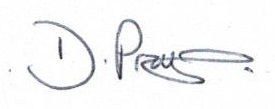
**

6^th^ November 2017

NB. Dr Przulj is also the Principal Investigator

# SUMMARY/SYNOPSIS

| **Short Title** | TRE |
| --- | --- |
| **Methodology** | Cohort Follow-up |
| **Research Sites** | 1. Queen Mary University of London  Health and Lifestyle Research Unit  2 Stayner’s Road  London  E1 4AH |
| **Objectives**  **/Aims** | Time-restricted eating (TRE) allows food intake during a period of eight hours each day. TRE showed promising results in animal studies and become popular with dieters. This is a pilot study to assess its acceptability and short-term effects by examining 1. Client adherence compared with other methods used with similar clients; 2. Weight loss compared with other methods used with similar clients; 3. Changes in heart risk factors; 4. Whether it would be worth conducting a bigger, controlled trial. |
| **Number of Participants/Patients** | 50 |
| **Main Inclusion Criteria** | - aged over 18 years, - BMI over 30kg/m2, or over 28kg/m2 with comorbidities - Speaks, reads, and understands English |
| **Statistical Methodology and Analysis (if applicable)** | Descriptive statistics will be used to report on the proportion of participants adhering to TRE; the 8-hour time frames most commonly selected; and client feedback ratings. Paired samples t-test will be used to examine any significant changes from baseline to follow-up in weight, BMI, blood pressure, and blood lipid profiles. Adherence rates to TRE and weight/BMI changes will be compared against previous weight-loss intervention cohorts (e.g. our previous trials, community weight clinic), via Chi-square analysis for categorical variables and ANOVA for continuous variables. |
| **Proposed Start Date** | Grant: November 2017  Recruitment: February 2018 |
| **Proposed End Date** | Recruitment: May 2018  Grant: November 2018 |
| **Study Duration** | 12 months (3m for set-up; 3m for recruitment; 3m for final follow-ups; 3m for write-up and study close) |

# INTRODUCTION

**Background**

Over a quarter of adults in England are obese, with obesity more prevalent in disadvantaged socioeconomic groups (1). Ill health resulting from obesity is considered to be responsible for about 10% of morbidity and mortality in the UK (2). The health burden from obesity is largely driven by an increased risk of type 2 diabetes, cancer and cardiovascular diseases (3).

Simple and effective weight loss interventions that are easy to disseminate are lacking. Intermittent fasting (IF) that involves intermittent periods of total or partial energy restriction alternated with non-restricted energy intake is one potentially promising approach.

There is preclinical evidence showing that in animals, regular periods of IF accompanied by no other food restrictions, generates weight loss and improvements in cardio-metabolic health (4–7). In these studies, IF generated a reduction in total cholesterol and triglycerides, an increase in high density lipoprotein (HDL) cholesterol and in some animal models, it reproduced some of the cardiovascular benefits seen with physical activity, such as improvements in blood pressure and heart rate and increased resistance to ischaemic injury. In humans, IF can generate weight loss and a range of associated benefits, with improvements in glucose metabolism and lipid profiles, particularly promising in terms of cardiovascular health (8–13).

In terms of clinical practice however, the approach has a notable limitation; the existing human studies typically used a rather harsh form of IF with fasting on two consecutive days and caloric restrictions during the rest of the week (14). Within tightly controlled trials, a proportion of clients are able to persist with this approach for the trial duration, but the approach is too difficult for most people and it is not used in clinical practice.

IF, however, can be also implemented in less severe formats, one of which is ‘Time-Restricted Eating’ (TRE) or ’16:8’ diet. TRE requires clients to consume food only within a specific ‘window’ period each day, typically over eight hours, and refrain from any eating outside of this window. The simplicity of the approach could be useful particularly to disadvantaged client groups that rarely engage with more intensive and demanding interventions (15).

In terms of evidence available so far and any pilot data, animal studies suggest that even in this much simplified format, TRE still conveys benefits (16). Some human data are now also available, coming from a somewhat non-traditional application. Due to its supposed effects on muscle mass and fat, TRE has become popular among athletes and body builders. Two recent small RCTs found some benefits in resistance-training men (17, 18). A recent conference presentation reported that TRE practiced by 11 participants over 4 days reduced swings in hunger and increased fat and protein oxidization (19).

This is a proposal to evaluate the acceptability and short-term effects of TRE in an inexpensive and quick pilot study focusing on clients from areas of high social deprivation who are most likely to benefit from this particular approach, compared to other diets that require calorie counting and much more extensive behaviour change.

# TRIAL OBJECTIVES

**Primary Objective**

The primary objective of the study is to determine the feasibility of the TRE approach by examining adherence to TRE and retention rates.

**Secondary Objectives**

Secondary objectives include TRE effects on changes in weight, blood

pressure and lipid profile, client feedback, and, provided the intervention is

feasible and the efficacy results are encouraging, effect estimates to inform

any future randomized trial of the intervention.

**Primary Outcome**

The primary outcome is the adherence to TRE over a period of 3 months, specifically the average number of days on which TRE was adhered to. We will also monitor the retention rates at 6 weeks and three months, defined as the proportion of participants who adhere to TRE at least once a week; and the drop-out rate defined as the proportion of participants who do not attend the final three-month follow-up session. We will compare retention and drop-out rates with those in our previous trials and community clinics.

**Secondary Outcomes**

- Changes in weight from baseline to 6 weeks, and to 3 months
- Changes in blood pressure from baseline to 3 months
- Changes in lipid profile from baseline to 3 months;
- Participant feedback and ratings of the intervention, including the helpfulness of the intervention, recommending it to friends, readiness to continue, barriers and facilitators, and most popular 8-hour time frames.
- Changes in ratings of the difficulty adhering to TRE and ratings of hunger over time
- Estimates of effect size for future randomised trials, if TRE is deemed feasible and efficacy is encouraging.
- Comparison of weight changes in TRE to weight changes in other weight-loss cohorts in our previous trials and community clinics.

# METHODOLOGY

**Inclusion Criteria**

- aged over 18 years,
- BMI over 30kg/m2, or over 28kg/m2 with comorbidities
- Speaks, reads, and understands English

**Exclusion Criteria**

- Medical condition precluding fasting, including a history of eating disorders;
- serious illness;
- currently on psychiatric medication;
- pregnancy or breastfeeding;
- has lost more than 5% of body weight in the last 6 months
- currently using TRE or another fasting approach to weight loss.

**Study Design**

Cohort follow-up.

**Study Scheme Diagram**

| **Recruitment**  Potential participants contact the study team via phone/email after seeing study ads and are screened. If eligible, they receive appointment and are sent the Participant Information Sheet (PIS). | | | | | | | |
| --- | --- | --- | --- | --- | --- | --- | --- |
|  |  | | |  | |  |  |
| **Baseline visit**  Informed consent; baseline questionnaires and measures of weight, BMI, blood pressure (BP) and lipid profile. TRE instructions given. | | | | | | | |
|  | |  |  |  |  | |  |
| **Week 1 Visit:** Participants will attend the clinic to give their weight and report on progress. | | | | | | | |
|  | | | |  | | | |
| **Weeks 2 to 5 Phone calls:** Participants will be called to check on their progress, and to collect data on self-reported weight and adherence. | | | | | | | |
|  | | | |  | | | |
| **Week 6 Visit:** Participants will attend the clinic to give their weight and report on their progress. | | | | | | | |
|  | | | |  | | | |
| **Week 12 visit:** Participants will attend the clinic to report on their progress. Measures of weight, BP, and limpid profile will be taken | | | | | | | |

# STUDY PROCEDURES

**Participant recruitment**

Participants will be recruited via the following methods; advertising in newspapers including the Evening Standard, Metro and local papers; adverts on social media and on internet fora; online bulletins; leaflets and posters in community venues; leaflets via mail drops; and through our community weight clinic.

Prospective participants will contact the study team by telephone or e-mail. They will be screened and if eligible, will receive an appointment for the baseline visit and PIS will be sent by post or e-mail.

**Baseline Visit**

At the baseline visit, after completing the consent procedures, eligible participants will complete study questionnaires (see details below), and measurements of weight, blood pressure and a blood sample for lipid profile will be collected. Participants will than receive an explanation of the TRE intervention and of the follow-up procedures and the TRE diary.

**TRE intervention**

Participants will be asked to consume all their food during an 8 hour period each day for the next three months. Over the remaining 16 hours each day, participants can drink, but only non-calorie drinks (e.g. water or coffee/tea with no milk or sugar). Participants will be free to choose which 8-hour period they would like to eat in.

**Follow-up visits and calls:** Participants will attend at one and six weeks, to provide measures of weight and to discuss their progress. They will also receive phone calls at weeks 2, 3, 4 and 5, that will monitor progress and collect data on adherence.

**Final follow-up visit**: Participants will attend at 12 weeks to provide measures of weight and blood pressure, and a blood sample for lipid profile and to report on their adherence to TRE and provide the final TRE ratings.

Participants will receive £10 at the 6 and 12 weeks visits as a compensation for their time and travel. Participants providing a blood sample at baseline and 12 weeks, will also receive £10 for each sample given.

**Measures**

***Baseline:***

- Demographic information collected via routine clinic questionnaires
- Weight, BMI
- Blood pressure
- Blood lipid profile
- Physical activity levels (IPAQ)

***Weekly:***

- Weight (Week 1 and 6 only)
- Number of days on which TRE was adhered to
- 8-hour time periods selected.
- Ratings of TRE helpfulness, TRE difficulty, ratings of hunger on days TRE was adhered to
- Comments on TRE barriers and facilitators (Week 1 and 6 only)

***Final follow-up at 12 weeks:***

- Weight
- Blood pressure
- Blood Lipid profile
- IPAQ
- TRE adherence
- TRE ratings (helpfulness, difficulty, ratings of hunger, would recommend TRE to friends, barriers and facilitators)

**Subject withdrawal**

Participants will be able to stop the TRE intervention at any time. Participants who stop the intervention will be followed up at 6 weeks and 3 months, unless they do not wish to be.

Unless withdrawn participants request otherwise, data collected up to the point of their withdrawal will be used in the study analysis. Withdrawn participants will not be replaced.

**Schedule of Assessment**

| **Study measures and Procedures** | **Time Point** | | | | | | | |
| --- | --- | --- | --- | --- | --- | --- | --- | --- |
|  | **Baseline** | **Wk1** | **Wk2** | **Wk3** | **Wk4** | **Wk5** | **Wk6** | **3m FU** |
| Consent | X |  |  |  |  |  |  |  |
| Screening | X |  |  |  |  |  |  |  |
| Demographics/medical history | X |  |  |  |  |  |  |  |
| IPAQ | X |  |  |  |  |  |  | X |
| Weight | X | X | X | X | X | X | X | X |
| BMI | X |  |  |  |  |  |  |  |
| Blood Lipid profile | X |  |  |  |  |  |  | X |
| Blood pressure | X |  |  |  |  |  |  | X |
| TRE instructions | X |  |  |  |  |  |  |  |
| Concurrent medication/comorbiidities | X |  |  |  |  |  |  | X |
| TRE Adherence |  | X | X | X | X | X | X | X |
| Feedback/ratings |  | X | X | X | X | X | X | X |

**End of Study Definition**

The study would be completed and the REC informed after the final attempt to collect 3-month follow-up data from the last participant.

# STATISTICAL CONSIDERATIONS

**Sample Size**

In this early phase exploratory research, we opted for a pragmatic sample size of 50, achievable economically and quickly, but large enough to provide reasonable confidence intervals on key estimates.

**Statistical Analysis**

Descriptive statistics will be used to report on proportions of participants adhering to TRE; the 8-hour time frames most commonly selected; and client feedback ratings. Paired samples t-test will be used to examine any significant changes from baseline to follow-up in weight, BMI, blood pressure, and blood lipid profiles.

Adherence rates to TRE and weight/BMI changes will be compared against previous weight-loss intervention cohorts (e.g. our previous trials, community weight clinic), via Chi-square analysis for categorical variables and ANOVA for continuous variables.

# ETHICS

The study will be carried out in accordance with the ethical principles in the Research Governance Framework for Health and Social Care, Second Edition, 2005 and its subsequent amendments as applicable and applicable legal and regulatory requirements.

This protocol and any subsequent amendments, along with any accompanying material provided to the participants and any advertising material will be submitted by the Chief Investigator to the sponsor and REC.

The CI will send the Annual Progress Report to the main REC and to the QMUL JRMO using the NRES template.

Participant identifiable data will remain confidential, and will be handled, processed, stored and destroyed according to the terms of the Data Protection Act 1998. All study data collected will be stored securely, and anonymised, and will not contain any identifying information. Only study staff and representatives of the sponsor or regulatory authorities (to the extent that they are allowed by law) will have potential access to view study data.

**Conflicts of Interest**

No applicant declares any conflict of interest.

# DATA HANDLING AND RECORD KEEPING

**Confidentiality**

Only study personnel and the study sponsor will have access to study data. We will not request any information about participants from their doctors.

Participants will not be identifiable from their study data. They will be assigned a participant ID number. Study data will be collected on paper CRFs, and will contain the participant ID only. Patient identifiable data will be entered onto a spreadsheet, for the purposes of contacting participants throughout the study. This data will be kept securely, and separately to the CRFs. The spreadsheet will be password protected and held on an encrypted USB stick. When not in use, the USB will be stored in a locked drawer. Original paper copies will be filed and kept in a locked cabinet. Only study staff will have access to the USB and paper copies.

All information will be kept confidential. Copies of all documents regarding the study will be kept in the trial master file (TMF) and/or relevant site file.

**Record Retention and Archiving**

All paper information relevant to the study will be archived and retained for 20 years at the Barts Health NHS Trust facility in Prescot Street. The sponsor will be informed in writing when and where all data is archived.

# LABORATORIES

**Central Laboratories**

Lipid profile analysis will be conducted by the Doctors Laboratory Ltd. (60 Whitfield Street, London W1T 4EU).

## Sample Collection, Labelling and Logging

Samples would be collected by trained phlebotomists in accordance with the Doctors Laboratory instructions, and labelled with participant ID number, time-point/session number, and date.

All samples taken will be logged.

**Sample Storage Procedure**

Samples will be temporarily stored at the Health and Lifestyle Research Unit (for up to 2 days maximum), at ambient room temperature, in a secure cabinet which only study staff will have access to.

## Sample Receipt

Samples will be sent to the central lab via their courier service. Upon receipt of the samples, the laboratory would ensure that the physical integrity of these samples have not been compromised in transit. If they have, the CI and sponsor will be informed of this. Upon receipt of samples, laboratory staff would also ensure that all samples are accounted for.

## Sample Analysis

A full Lipid profile will be obtained, including Total Cholesterol, Triglycerides and HDL Cholesterol. Total Cholesterol and Triglycerides will be analysed using the enzymatic, colorimetric method; and HDL Cholesterol with the homogeneous enzymatic colorimetric test.

**Sample destruction**

When all the samples have been analysed and the data has been entered, the samples will be destroyed by the Doctors Laboratory in accordance with the Human Tissue Authority’s Code of Practice.

# MONITORING & AUDITING

Monitoring will be proportional to the objective, scope, design, size, complexity and risks of the project. QMUL JRMO will risk assess the trial in line with the QMUL JRMO risk assessment SOP. The trial’s risk assessment will be used by the Study Manager and CI/PI to create a monitoring plan (detailing the type, duration and frequency of monitoring). A Copy of the plan will be kept in the TMF. The CI will ensure that the agreement/wording is not altered without written authorisation (email confirmation) from the QMUL JRMO; all new versions will be signed.

CI/study team will notify the QMUL JRMO’s GCP team once the first patient has been consented. .

# TRIAL COMMITTEES

A Trial Management Group (TMG), consisting of the CI, study manager and other key study staff, will meet every month during the recruitment phase, to oversee the running of the project**.**

# FINANCE AND FUNDING

The Study is funded by a Project Grant from British Heart Foundation (£61,574).

# INDEMNITY

The study Sponsor in the UK will be QMUL.QMUL JRMO has arranged for suitable indemnity concerning negligent harm to be in place for the study in the UK.

The insurance that QMUL has in place provides "No Fault Compensation" for participants which provides an indemnity to participants for non-negligent harm.

# DISSEMINATION OF RESEARCH FINDINGS

Study results would be:

(1) Communicated to the BHF

(2) Published in Open Access format in a prominent journal.

(3) Presented at a conference on obesity

# REFERENCES

1. Health and Social Care Information Centre. Statistics on Obesity, Physical Activity and Diet - England, 2015 [Internet]. 2015 Mar [cited 2015 Jul 23]. Available from: <http://www.hscic.gov.uk/catalogue/PUB16988>

2. Rayner M, Scarborough P. The burden of food related ill health in the UK. J Epidemiol Community Health. 2005 Jan 12;59(12):1054–7.

3. Wang YC, McPherson K, Marsh T, Gortmaker SL, Brown M. Health and economic burden of the projected obesity trends in the USA and the UK. The Lancet. 2011 Aug 27;378(9793):815–25.

4. Rothschild J, Hoddy KK, Jambazian P, Varady KA. Time-restricted feeding and risk of metabolic disease: a review of human and animal studies. Nutr Rev. 2014 May;72(5):308–18.

5. Mattson MP, Duan W, Guo Z. Meal size and frequency affect neuronal plasticity and vulnerability to disease: cellular and molecular mechanisms. J Neurochem. 2003 Feb;84(3):417–31.

6. Halagappa VKM, Guo Z, Pearson M, Matsuoka Y, Cutler RG, Laferla FM, et al. Intermittent fasting and caloric restriction ameliorate age-related behavioral deficits in the triple-transgenic mouse model of Alzheimer’s disease. Neurobiol Dis. 2007 Apr;26(1):212–20.

7. Hatori M, Vollmers C, Zarrinpar A, DiTacchio L, Bushong EA, Gill S, et al. Time-Restricted Feeding without Reducing Caloric Intake Prevents Metabolic Diseases in Mice Fed a High-Fat Diet. Cell Metab. 2012 Jun 6;15(6):848–60.

8. Barnosky AR, Hoddy KK, Unterman TG, Varady KA. Intermittent fasting vs daily calorie restriction for type 2 diabetes prevention: a review of human findings. Transl Res. 2014 Oct;164(4):302–11.

9. Varady KA. Intermittent versus daily calorie restriction: which diet regimen is more effective for weight loss? Obes Rev Off J Int Assoc Study Obes. 2011 Jul;12(7):e593-601.

10. Harvie MN, Pegington M, Mattson MP, Frystyk J, Dillon B, Evans G, et al. The effects of intermittent or continuous energy restriction on weight loss and metabolic disease risk markers: a randomized trial in young overweight women. Int J Obes 2005. 2011 May;35(5):714–27.

11. Patterson RE, Laughlin GA, LaCroix AZ, Hartman SJ, Natarajan L, Senger CM, et al. Intermittent Fasting and Human Metabolic Health. J Acad Nutr Diet. 2015 Aug;115(8):1203–12.

12. Varady KA, Bhutani S, Church EC, Klempel MC. Short-term modified alternate-day fasting: a novel dietary strategy for weight loss and cardioprotection in obese adults. Am J Clin Nutr. 2009 Nov;90(5):1138–43.

13. St-Onge M-P, Ard J, Baskin ML, Chiuve SE, Johnson HM, Kris-Etherton P, et al. Meal Timing and Frequency: Implications for Cardiovascular Disease Prevention: A Scientific Statement From the American Heart Association. Circulation. 2017 Jan 1;CIR.0000000000000476.

14. Williams KV, Mullen ML, Kelley DE, Wing RR. The effect of short periods of caloric restriction on weight loss and glycemic control in type 2 diabetes. Diabetes Care. 1998 Jan;21(1):2–8.

15. Harvey JR, Ogden D. Obesity Treatment in Disadvantaged Population Groups: Where Do We Stand and What Can We Do? Prev Med. 2014 Nov;68:71–5.

16. Chaix A, Zarrinpar A, Miu P, Panda S. Time-restricted feeding is a preventative and therapeutic intervention against diverse nutritional challenges. Cell Metab. 2014 Dec 2;20(6):991–1005.

17. Tinsley GM, Forsse JS, Butler NK, Paoli A, Bane AA, La Bounty PM, et al. Time-restricted feeding in young men performing resistance training: A randomized controlled trial. Eur J Sport Sci. 2017 Mar;17(2):200–7.

18. Moro T, Tinsley G, Bianco A, Marcolin G, Pacelli QF, Battaglia G, et al. Effects of eight weeks of time-restricted feeding (16/8) on basal metabolism, maximal strength, body composition, inflammation, and cardiovascular risk factors in resistance-trained males. J Transl Med [Internet]. 2016 Oct 13 [cited 2017 Feb 26];14. Available from: <http://www.ncbi.nlm.nih.gov/pmc/articles/PMC5064803/>

19. Peterson C. Time-Restricted Feeding Increases Fat Oxidation and Reduces Swings in Appetite Levels in Humans. Oral Abstract Presentation presented at: The Obesity Society Annual Meeting at Obesity Week 2016; 2016 Nov 31; Pennington Biomedical Research Center.
